# Metformin treatment is associated with improved outcome in patients with diabetes and advanced heart failure (HFrEF)

**DOI:** 10.1038/s41598-022-17327-4

**Published:** 2022-07-29

**Authors:** Jan Benes, Martin Kotrc, Katerina Kroupova, Peter Wohlfahrt, Jan Kovar, Janka Franekova, Marketa Hegarova, Lenka Hoskova, Eva Hoskova, Terezie Pelikanova, Petr Jarolim, Josef Kautzner, Vojtech Melenovsky

**Affiliations:** 1 Department of Cardiology, Institute for Clinical and Experimental Medicine-IKEM, Videnska 1958/9, 140 21 Praha 4, Czech Republic; 2 Department of Pathology, Brigham and Women’s Hospital, Harvard Medical School, Boston, MA USA

**Keywords:** Cardiology, Heart failure, Diabetes

## Abstract

The role of metformin (MET) in the treatment of patients with advanced HFrEF and type 2 diabetes mellitus (DM) is not firmly established. We studied the impact of MET on metabolic profile, quality of life (QoL) and survival in these patients. A total of 847 stable patients with advanced HFrEF (57.4 ± 11.3 years, 67.7% NYHA III/IV, LVEF 23.6 ± 5.8%) underwent clinical and laboratory evaluation and were prospectively followed for a median of 1126 (IQRs 410; 1781) days for occurrence of death, urgent heart transplantation or mechanical circulatory support implantation. A subgroup of 380 patients (44.9%) had DM, 87 of DM patients (22.9%) were treated with MET. Despite worse insulin sensitivity and more severe DM (higher BMI, HbA1c, worse insulin resistance), MET-treated patients exhibited more stable HF marked by lower BNP level (400 vs. 642 ng/l), better LV and RV function, lower mitral and tricuspid regurgitation severity, were using smaller doses of diuretics (all p < 0.05). Further, they had higher eGFR (69.23 vs. 63.34 ml/min/1.73 m^2^) and better QoL (MLHFQ: 36 vs. 48 points, p = 0.002). Compared to diabetics treated with other glucose-lowering agents, MET-treated patients had better event-free survival even after adjustment for BNP, BMI and eGFR (p = 0.035). Propensity score-matched analysis with 17 covariates yielded 81 pairs of patients and showed a significantly better survival for MET-treated subgroup (p = 0.01). MET treatment in patients with advanced HFrEF and DM is associated with improved outcome by mechanisms beyond the improvement of blood glucose control.

## Introduction

Type 2 diabetes mellitus (DM) is a common and severe comorbidity in patients with heart failure with reduced ejection fraction (HFrEF), but optimal treatment modality has not yet been clarified. Biguanides including metformin (MET) had long been considered contraindicated in HF patients due to concerns about lactic acidosis, that was observed with phenformin, an older biguanide with less favorable pharmacological profile^1^. Large meta-analysis, however, has not demonstrated an association between MET therapy and increased risk of lactic acidosis^2^, so MET has been used even in HF population. Observational studies showed not only MET safety in HF subjects^3–5^ but some studies even suggested a survival benefit associated with this drug^6–8^. However, there is only one study analyzing MET specifically in patients with HFrEF^9^ and the absence of a randomized trial is a major limitation for MET use. Moreover, registry-based retrospective studies lack a precise characterization of analyzed patients (echocardiography, laboratory analysis including metabolic profile). Therefore, the mechanism of MET action in this population is speculative.

The aim of the present study was to evaluate the association between MET treatment and metabolic profile, quality of life and outcome in prospectively followed advanced HFrEF patients.

## Methods

### Patients

Patients with stable HFrEF (LVEF < 40%) of least 6-month duration receiving a stable medication for at least 3 months were enrolled in the study between 2008 and 2016 in a prospectively defined registry. Subjects with potentially reversible LV dysfunction (planned valve surgery, revascularization, or tachycardia-induced cardiomyopathy) were excluded. Patients were followed until July 2019. DM was diagnosed according to current recommendation^10^. The investigation conforms with the principles outlined in the Declaration of Helsinki, the study protocol was approved by the Institutional Ethics Committee and all subjects signed an informed consent. At the study enrollment, patients completed a Minnesota Living with Heart Failure Questionnaire (MLHFQ) and had anthropometric tests and underwent an echocardiographic study (Vivid-7; General Electric, Milwaukee, Wisconsin). LV function and dimensions were measured according to recommendations^11^. RV dysfunction was quantified in four grades (0–3). Mitral and tricuspid regurgitations were assessed semiquantitatively and expressed in 3 grades (mild, moderate, significant). An adverse outcome was defined as the combined endpoint of death, urgent heart transplantation (HTx) or mechanical circulatory support (MCS) implantation^12^. Patients who received a non-urgent HTx were censored as having no adverse event at the day of HTx.

### Statistical analysis

Data are presented as mean ± standard deviation, median with interquartile ranges (IQRs), or frequency (percent). Unpaired t-test or Mann–Whitney test were used to compare continuous variables between groups as appropriate. The effect of biomarker concentration on prognosis was tested using univariate and multivariable Cox model. Event-free survival of patients was analyzed by Kaplan–Meier analysis with log-rank test comparison between groups. Propensity score matching was used to account for differences in characteristics of patients with and without MET. The propensity score for each patient was calculated using a multivariable logistic regression model in which the MET use was regressed on 17 characteristics (see “Results” section) that might influence the selection of MET therapy or that have been shown to influence prognosis of patients with advanced HF. All tests were 2-sided, and p values < 0.05 were considered significant. Calculations were performed using JMP 11 (SAS Institute Inc., Cary, NC) and R (Vienna, Austria). Methods in detail can be found in the Online Supplement.

### Ethics approval and consent to participate

The ethical committee of the Institute for Clinical and Experimental Medicine-IKEM and Thomayer hospital in Prague approved the study protocol. Written, informed consent for participation in the study was obtained from all the subjects. The study was performed in accordance with the Helsinki Declaration of 1964, and its later amendments.

## Results

### Patients

**Figure 1:**
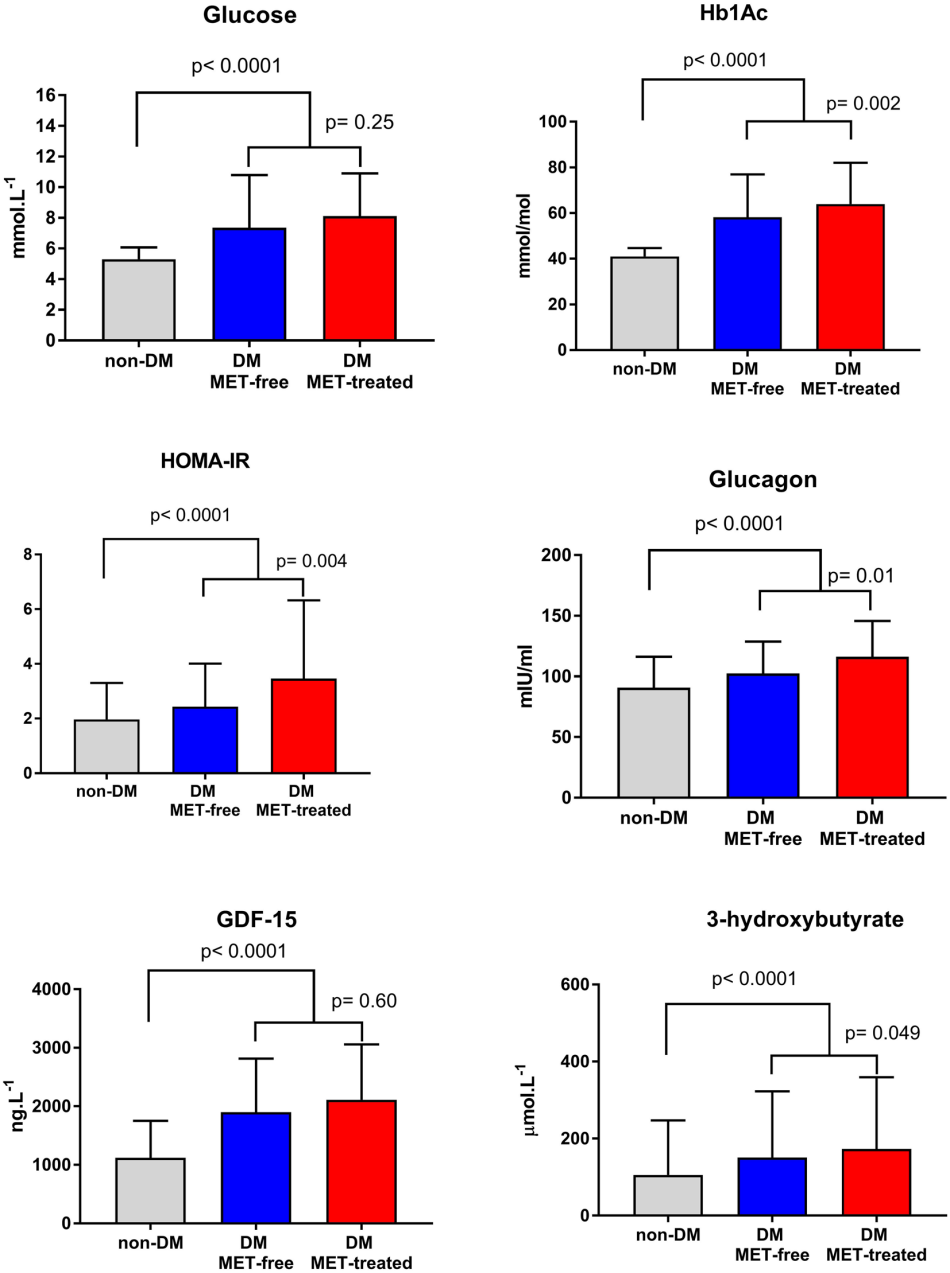
Metabolic profile. For Glucose and Hb1Ac, data are shown as mean ± SD, for HOMA-IR, Glucagon, GDF-15 and 3-hydroxybutyrate as median ± IQRs. For HOMA-IR, only patients without insulin treatment were evaluated (n = 196 DM MET-free, n = 61 DM MET-treated).

A total of 847 advanced HFrEF patients (67.7% were in with NYHA III/IV, average LV-ejection fraction was 23.6%, 44.9% had moderate/severe RV dysfunction), were enrolled in the study (Fig. 1 in the Online supplement). Enrolled patients achieved high level of guideline-recommended HF pharmacotherapy and device therapy (Table 1). Patients were prospectively followed for a median of 1126 (IQRs 410; 1781) days. During follow-up, 515 patients (60.8%) experienced an adverse outcome.

**Table 1:** Patients characteristics.

	Whole cohort (n = 847)	Non-DM (n = 467)	DM (n = 380)	P (non-DM vs. DM)	DM MET-free (n = 290)	DM MET-treated (n = 87)	P (MET-free vs. MET-treated)
Age (years)	57.40 ± 11.28	55.03 ± 11.94	60.31 ± 9.65	** < 0.0001**	60.14 ± 9.63	60.92 ± 9.85	0.51
Males (%)	82.8	81.6	84.2	0.31	83.5	86.2	0.53
HF etiology (% CAD)	50.2	41.6	60.8	** < 0.0001**	59.4	65.1	0.34
BMI (kg/m^2^)	27.82 ± 5.09	26.94 ± 4.55	28.9 ± 5.50	** < 0.0001**	28.27 ± 5.31	30.98 ± 5.61	** < 0.0001**
NYHA (2–4, %)	32.2/60.3/7.4	35.1/58.9/6.0	28.7/62.1/9.2	0.11	25.5/63.5/11.0	40.2/56.3/3.5	**0.02**
BNP (ng/l)	466 (208; 1077)	381 (162; 948)	613 (264; 1187)	** < 0.0001**	642 (334; 1354)	400 (148; 920)	**0.0002**
Hemoglobin (g/l)	140.85 ± 18.18	142.00 ± 18.36	139.49 ± 17.90	**0.049**	140.09 ± 18.13	138.00 ± 16.39	0.34
eGFR (ml/min 1.73/m^2^)	68.91 ± 22.50	72.55 ± 22.55	64.59 ± 21.69	** < 0.0001**	63.34 ± 22.12	69.26 ± 19.76	**0.03**
CRP (mg/l)	4.5 (1.9; 9.9)	3.5 (1.5; 8.1)	5.5 (2.5; 11.4)	** < 0.0001**	5.5 (2.9; 11.3)	5.3 (2.1; 13.2)	0.70
**Diabetes and metabolism**							
Glucagon (mIU/ml)	97 (77; 125)	90.8 (73.83; 116.2)	105.7 (82.5; 132.8)	** < 0.0001**	102.35 (80.35; 128.73)	116.30 (92.10; 145.70)	**0.015**
C-peptid (nmol/l)	1.38 (0.958; 1.942)	1.27 (0.92; 1.76)	1.52 (1.03; 2.16)	** < 0.0001**	1.50 (1.02; 2.12)	1.56 (1.08; 2.26)	0.90
Free fatty acids (mmol/l)	0.53 (0.37; 0.72)	0.49 (0.35; 0.69)	0.59 (0.40; 0.79)	**0.0008**	0.61 (0.39; 0.80)	0.57 (0.42; 0.79)	0.74
**Biomarkers**							
Hs-TnT^&^ (ng/l)	23.86 (14.46; 40.72)	20.05 (11.98; 33.41)	28.18 (18.71; 49.03)	** < 0.0001**	29.3 (18.8; 49.2)	25.3 (17.6; 43.1)	0.49
**Cardiac morphology and function**							
SBP (mmHg)	116.33 ± 19.10	115.3 ± 19.2	117.6 ± 18.9	0.07	116.08 ± 18.89	122.78 ± 18.00	**0.004**
Heart rate (min^−1^)	75.72 ± 14.54	74.22 ± 14.77	77.55 ± 14.07	**0.002**	77.66 ± 14.26	77.43 ± 13.61	0.90
LVEDD (mm)	69.41 ± 9.11	69.82 ± 9.72	68.90 ± 8.28	0.14	69.04 ± 8.47	68.31 ± 7.68	0.47
LVEF (%)	23.59 ± 5.80	23.57 ± 5.80	23.63 ± 5.79	0.88	23.05 ± 5.82	25.60 ± 5.30	**0.0003**
RVD1 (mm)	40.62 ± 7.94	39.64 ± 8.11	41.81 ± 7.58	** < 0.0001**	41.93 ± 7.81	41.40 ± 6.89	0.57
RV dysfunction grade (0–3, %)	32.3 22.8/33.5/11.4	38.5/23.8/28.6/9.1	24.7/21.6/39.5/14.2	** < 0.0001**	19.9/21.4/42.8/15.9	42.2/21.7/27.7/8.4	**0.002**
Mitral regurgitation (1–3, %)	25.2/40.5/34.3	27.0/40.3/32.7	22.9/40.8/36.3	0.32	18.3/44.1/37.6	39.1/28.7/32.2	**0.0004**
Tricuspid regurgitation (1–3, %)	44.6/39.0/16.3	48.9/37.4/13.7	39.4/41.0/19.6	**0.001**	34.0/44.5/21.5	57.5/31.0/11.5	**0.0004**
Estimated systolic pulmonary pressure (mmHg)	45.11 ± 13.65	43.46 ± 13.93	46.98 ± 13.10	**0.002**	46.97 ± 12.95	47.60 ± 13.05	0.74
IVC (mm)	19.55 ± 5.74	18.93 ± 5.58	20.31 ± 5.86	**0.0006**	20.45 ± 5.81	19.54 ± 5.36	0.21
**Quality of life**							
MLHFQ sum	44 (26; 60)	43 (24; 59)	44 (28; 61)	0.25	48 (30; 62)	36 (16; 51)	**0.002**
MLHFQ somatic	21 (12; 28)	21 (12; 27)	22 (12; 28)	0.59	23 (14; 29)	17 (10; 24)	**0.0007**
MLHFQ emotional	6 (2; 11)	6 (1; 12)	6 (2; 11)	0.86	6 (3; 12)	5 (1; 10)	0.09
**Hemodynamics**^**∆**^							
RA pressure (mmHg)	9 (6; 13)	8 (5; 12)	10 (6; 16)	**0.0009**	10 (6; 16)	9 (7; 14)	0.56
Systolic PA pressure (mmHg)	53 (38; 65)	47 (34; 62)	57 (44; 68)	** < 0.0001**	57 (44; 69)	60 (45; 68)	0.89
Diastolic PA pressure (mmHg)	24 (18; 31)	22 (16; 29.5)	27 (20; 32)	**0.005**	27 (19.5; 32)	26.5 (19.75; 32.25)	0.92
Mean PA pressure (mmHg)	35 (26; 43)	32 (23; 42)	37 (30; 45)	**0.0003**	37 (30; 45)	38 (32; 44)	0.99
PCWP (mmHg)	24 (17; 29)	23 (16; 28)	25 (19; 30)	**0.02**	24.5 (19; 30	25 (16; 31)	0.73
CI (l/min/1.73 m^2^)	1.84 (1.58; 2.15)	1.90 (1.59; 2.18)	1.80 (1.55; 2.14)	0.14	1.75 (1.51; 2.13)	1.96 (1.73; 2.32)	0.06
**Therapy**							
ACEi/ARB (%)	78.65	79.83	77.31	0.37	77.51	79.31	0.72
BB (%)	87.66	88.20	87.07	0.62	88.58	82.76	0.17
MRA (%)	76.99	75.32	78.89	0.22	79.93	75.86	0.42
Furosemide daily dose (mg)	80 (40; 125)	60 (40; 120)	80 (40; 125)	** < 0.0001**	80 (40; 131.25)	60 (40; 125)	**0.03**
ICD any (%)	59.4	57.8	61.4	0.29	61.70	61.45	0.97
CRT any (%)	32.0	31.5	32.6	0.73	35.11	24.10	0.06
Amiodarone (%)	18.3	17.9	18.8	0.73	19.66	16.09	0.45
Insulin (%)	–	–	28.4	–	30.34	22.99	0.18
Insulin daily dose	–	–	48 (31; 66)*	–	46 (31; 63.5)	54.5 (31.5; 80)	0.29
SU derivatives (%)	–	–	17.6	–	15.17	26.44	**0.02**
DPP-IV inhibitors (%)	–	–	6.8	–	4.83	13.79	**0.007**
**Outcome**							
Death (%)	324 (38.3%)	134 (28.7%)	190 (50.0%)	–	152 (52.4%)	37 (42.5%)	–
Urg. HTx (%)	107 (12.6%)	63 (13.5%)	44 (11.6%)	–	39 (13.5%)	4 (4.6%)	–
Norm. HTx (%)	35 (4.1%)	23 (4.9%)	12 (3.2%)	–	10 (3.5%)	2 (2.3%)	–
MCSi (%)	83 (9.8%)	48 (10.3%)	35 (9.2%)	–	28 (9.7%)	6 (6.9%)	–

Data are shown as mean ± SD or median with IQRs.

^&^Available in 450 patients only.

^∆^Available in 385 patients only.

*Calculated for only 108 patients treated with insulin. Information about DM treatment was missing in 3 patients.

Significant values are in bold.

**Figure 2:**
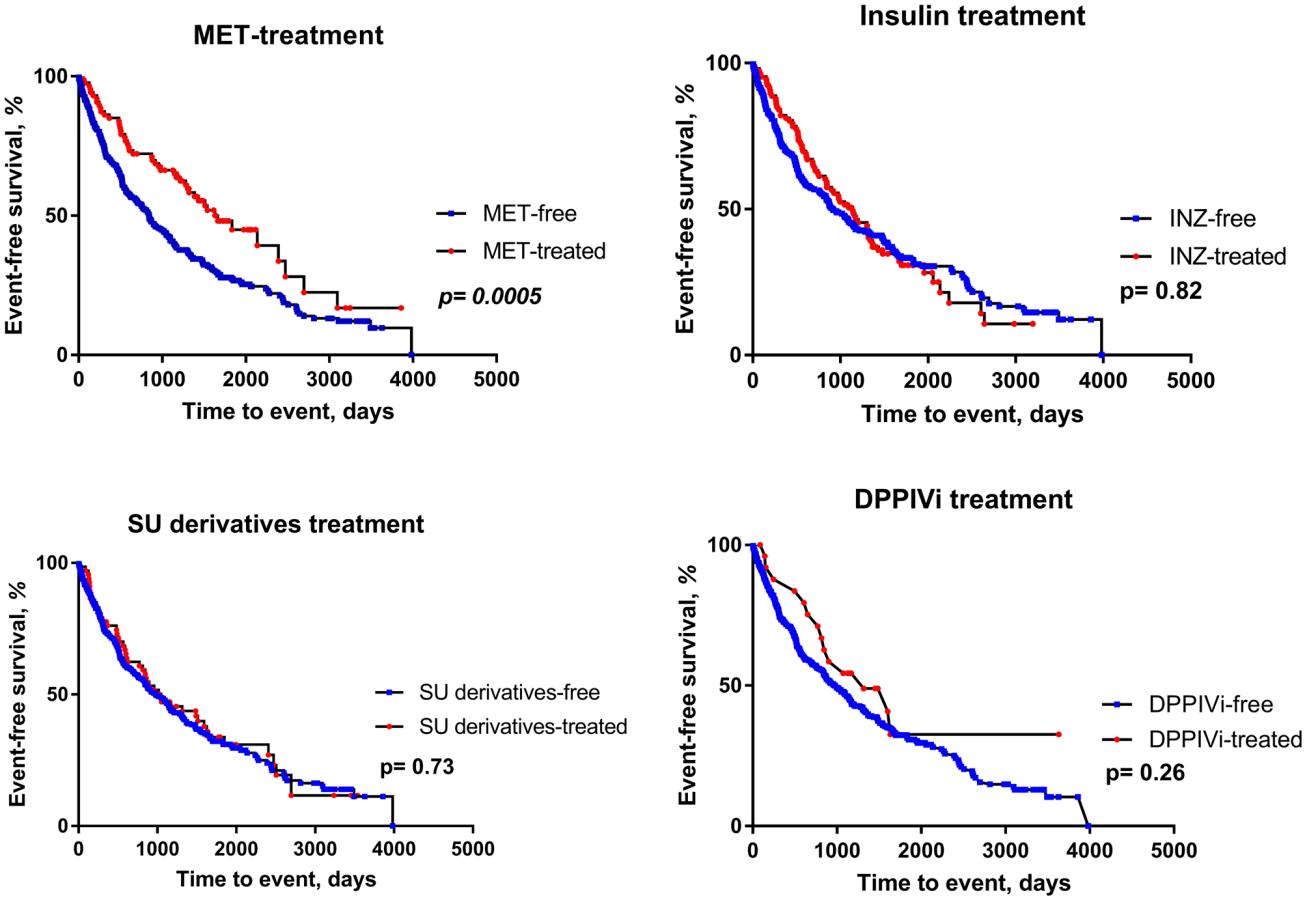
Event-free survival DM patients according DM treatment; MET-treated DM patients had significantly better survival, no significant difference in survival was observed among patients treated with other glucose-lowering agents.

A total of 380 patients (44.9%) were found to have DM, 467 patients (55.1%) were DM free. All DM patients had type 2 DM; none of the patients had type 1 DM. DM patients were older, had more often CAD as underlying HF etiology, larger body mass index, worse renal function (Table 1) and worse cumulative survival—269 (70.8%) DM vs. 246 (52.7%) non-DM patients experienced an adverse outcome, median time to event was 879 days (IQRs 312; 1631) for DM patients compared with 1270 (IQRs 467; 2010) days for non-DM counterparts. Kaplan–Meier curves are provided in Fig. 2 in the Online Supplement.

### Diabetes treatment

**Figure 3:**
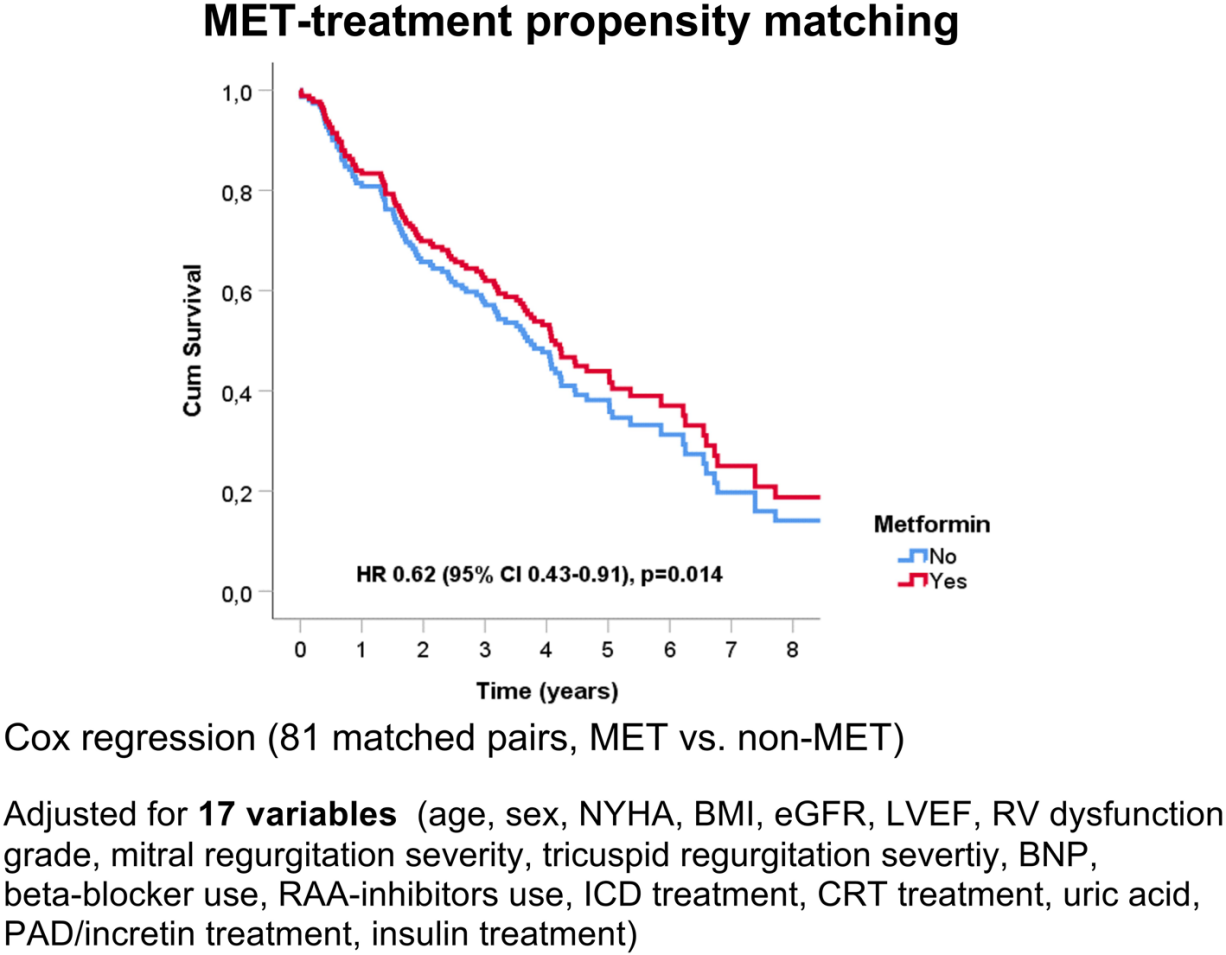
Survival of MET-treated patients, propensity-score matched analysis. *BMI* body mass index, *eGFR* estimated glomerular filtration rate, *LVEF* left ventricle ejection fraction, *RV* right ventricle, *BNP* B-type natriuretic peptide, *RAAi* renin-angiotensin system inhibitors, *ICD* implantable cardioverter/defibrillator, *CRT* cardiac resynchronization, therapy, *PAD* peroral antidiabetics.

Out of 380 DM patients, 153 patients (40.3%) were treated with diet only, 87 patients (22.9%) with MET, 67 patients (17.6%) with sulfonylurea (SU) derivatives, 108 patients (28.4%) with insulin, 26 patients (6.8%) with DPPIV-inhibitors, 3 patients (0.8%) with repaglinide and 1 patient (0.3%) was treated with liraglutide. In 3 patients the information about the treatment was missing. None of the patients was treated with thiazolidinediones, acarbose or SGLT2-inhibitors. 31 patients (8.2%) were treated with more than one peroral antidiabetics (PAD), 26 patients (6.8%) with the combination of PAD and insulin. More detailed information about DM treatment is given in Table 1 and Fig. 3 in the Online Supplement.

In patients treated with MET, the most widely used MET dose was 1000 mg (29 patients, 33.3%). 18 patients (20.7%) were taking a dose lower than 1000 mg, 14 patients (16.1%) a dose between 1000 and 2000 mg and 25 patients (28.8%) were taking 2000 mg daily or higher. The information about MET daily dose was missing in 1 patient (1.1%). Distribution of MET daily dose is in Fig. 4 in the Online Supplement.

Compared with MET-free counterparts, MET-treated DM patients had better LV function (LVEF), RV function and lower both mitral and tricuspid regurgitation severity, better renal function and larger BMI. They were using smaller diuretic doses but achieved similar level of guideline-recommended HF pharmacotherapy, had comparable rate of ICD and CRT treatment and similar hemodynamic profile (Table 1). MET-treated patients were more often treated with SU derivatives and DPPIV-inhibitors; no significant difference was found for insulin treatment.

### Metabolic profile of MET-treated patients

Analysis of metabolic parameters revealed that compared with MET-free counterparts, MET-treated patients had similar levels of fasting glycemia and insulin secretion (C-peptide level), but larger Hb1Ac level, higher insulin and glucagon level and more pronounced insulin resistance (HOMA-IR), Table 1 and Fig. 1. Further, MET-treated patients had higher level of beta-hydroxybutyrate but similar level of GDF-15 (Fig. 1).

### Diabetes treatment and quality of life

No significant difference was found in QoL between patients with and without DM (Table 1). In DM subgroup, pharmacotherapy with neither insulin, SU derivatives nor with DPPIV-inhibitors was associated with better QoL (Table 1 in the Online Supplement). On the contrary, MET treatment was associated with a better QoL (Table 1).

Multivariable regression analysis identified MET treatment together with BNP and BMI, but not eGFR, SU derivatives treatment, DPPIV-inhibitors treatment or insulin treatment to be independently associated with MLHFQ score (Table 2 in the Online Supplement). Similar results were obtained for the somatic component of MLHFQ whereas no association of MET treatment with emotional component of the MLHFQ score was found (data not shown).

### Diabetes treatment and outcome

Kaplan–Meier analysis showed that MET-treated diabetic patients had better survival compared to MET-free counterparts. Other therapeutic regimes were not associated with any difference in event-free survival (Fig. 2). Similarly, Cox proportional hazard model identified MET treatment to be associated with improved outcome. No such relationship was observed for therapy with insulin, SU derivatives or DPPIV-inhibitors (Table 3 in the Online Supplement).

Next, we have analyzed whether there was any subgroup having altered benefit from MET treatment. No significant interaction was found between MET therapy and NYHA functional class, LVEF, RV dysfunction grade, BNP level, eGFR, ACEi/ARB treatment, beta-blocker treatment, presence of ICD, or CRT (all p for interaction ≥ 0.20, Table 4 in the Online Supplement). This suggests that the benefit from MET therapy is preserved regardless of HF severity and independent of HF treatment. Similarly, no significant interaction was found between MET therapy and insulin or DPPIVi treatment (p for interaction = 0.35 and 0.95, respectively). However, borderline interaction was found for SU derivatives treatment (p for interaction = 0.054). Kaplan–Meier analysis showed borderline worse survival of patients treated with MET and SU derivatives compared with MET without SU derivatives (p (log-rank) = 0.08).

### Adjustment for confounders, propensity score-matched analysis

Although MET-treated DM patients had better cardiac function, renal function and larger BMI, Cox proportional hazard model analysis revealed that MET treatment was associated with a significantly better outcome even after the adjustment for BNP, eGFR and BMI (Table 2).

**Table 2:** Metformin and outcome, Cox proportional hazard analysis.

		HR	95% CI	p
Model 1	MET (present vs. absent)	0.57	0.41–0.78	**0.0003**
Model 2	MET (present vs. absent)	0.63	0.45–0.87	**0.004**
	BMI (kg/m^2^)	0.97	0.94–0.99	**0.005**
Model 3	MET (present vs. absent)	0.64	0.46–0.88	**0.007**
	BMI (kg/m^2^)	0.97	0.94–0.99	**0.01**
	eGFR (ml/min 1.73/m^2^)	0.995	0.989–1.0006	0.08
Model 4	MET (present vs. absent)	0.70	0.50–0.98	**0.035**
	BNP (ng/l)	1.00056	1.0004–1.0007	** < 0.0001**
	BMI (kg/m^2^)	0.99	0.97–1.018	0.51
	eGFR (ml/min 1.73/m^2^)	0.996	0.991–1.002	0.24

MET treatment was associated with lower risk of an adverse outcome even after the adjustment for BNP, eGFR and BMI.

*HR* hazard ratio, *CI* confidence interval.

Significant values are in bold.

Finally, we have performed propensity score matched analysis that matched the patients for 17 variables that might influence the selection of MET therapy or that have been shown to influence prognosis of patients with advanced heart failure (age, sex, NYHA functional class, BMI, estimated glomerular filtration rate, LVEF, RV dysfunction grade, mitral and tricuspid regurgitation severity, BNP level, beta-blockers use, renin-angiotensin system inhibitors use, ICD therapy, CRT therapy, uric acid levels, treatment with other PAD/incretins and treatment with insulin). Propensity score matching yielded 81 pairs of patients. Standardized mean differences of matched covariates ranged from 0 to 0.23, with a standardized median difference of 0.06 (IQR 0.029–0.076). Significantly better survival for MET-treated group was showed both using the McNemar (p = 0.04), as well as Cox proportional hazard model (p = 0.01, Fig. 3).

## Discussion

The results of this study can be summarized as follows: (i) despite worse insulin sensitivity and worse DM compensation in MET-treated patients, MET-treatment was independently associated with both better quality of life and improved outcome in advanced HFrEF patients with DM; (ii) MET treatment was associated with better outcome regardless of HF severity or compensation of diabetes.

Optimal treatment modality in patients with advanced HF and DM is not well established, which is mirrored by the large variability of treatment strategies observed in our study.

Only 22.9% of our patients were treated with MET, which is likely a consequence of previous recommendations to avoid this drug in HF because of concerns regarding lactic acidosis risk^13^. Nevertheless, MET was used in clinical practice and data on MET use in HF patients with DM eventually emerged. One recently published study showed lower risk of hospitalization for HF in MET-treated DM patients^14^. Thirteen studies have been published describing the association between MET treatment on outcome in patients with established HF and DM^6,7,9,15–24^. However, the majority of studies are retrospective and based on administrative or disease records^7,17–21,23,24^. Only five of them reported LV-ejection fraction^6,7,9,17,22^ and only one study focused specifically on patients with LVEF < 40%^9^. Although meta-analyses of these studies reported mostly better outcome in patients treated with MET^3,4^, the heterogeneity of studied populations and approaches leave many questions unanswered. None of the studies focused specifically on patients with advanced HF and no study HFrEF patients employed propensity-matching approach. As large randomized controlled trials with MET in HF patients with DM are unlikely to be carried out^25^, our data offering a prospective observational design of well-characterized cohort employing propensity matching analysis offers the strongest evidence possible. In the propensity matching analysis, we have adjusted the cohort for seventeen possible confounders and our data thus strongly suggest that despite differences between MET-treated and MET-free patients, observed difference in outcome between these groups is indeed attributable to MET therapy.

The mechanism of action of MET is a subject of intense debate. Beneficial effect of MET was first explained by an inhibition of mitochondrial complex I^26,27^ and by an increase in ADP/ATP ratio that activates AMP-dependent protein kinase (AMPK). However, the mechanism of action of MET is likely to be more pleiotropic; MET enhances cardiac autophagy^28^, improves myocardial efficiency and reduces myocardial energy consumption^29^, and directly modulates the growth and function of gut microbiota^30^. MET has been also shown to have potent effect on cancer prevention and recurrence^31,32^ and its anti-cancer effect might be also clinically relevant in HF patients as they are consistently reported to have higher risk of malignancies^33,34^.

Although our study was not designed to unveil the mechanism responsible for overall benefit from MET therapy, our data suggest that the cardioprotection of MET is independent on glycemic control. This is in line with results of the post UKPDS-trial follow-up that showed significant risk reduction by MET in diabetes-related endpoints despite of the loss of between-group differences in glycated hemoglobin^35^. Experimental studies have similarly shown anti-inflammatory properties of MET irrespective of DM status^36^.

Metabolic abnormalities are observed early in the course of cardiac pathologies. When subjected to pressure overload, the ventricular myocardium shifts from fatty acids to glucose as its main source for energy; this precedes the development of LV hypertrophy^36^. The excessive glucose metabolism in the cardiomyocytes causes glucose-6-phosphate (G6P) accumulation. G6P activates mammalian target of rapamycin complex 1 (mTORC1), which induces hypertrophy. MET activates AMPK, which inhibits mTROC1, thus preventing LV hypertrophy^36^. This explains that MET treatment has also been shown to induce regression of LV-hypertrophy and exert anti-oxidant effects even in non-diabetic patients^37^. Similarly, LV reverse remodeling has been observed in other drugs that activate AMPK such as SGLT2-inhibitors^38^.

In non-HF subjects, MET was shown to mediate its effect on body weight and energy balance through GDF-15^39^. We have not observed increased GDF-15 level in MET-treated compared with MET-free patients in our study, which can be explained by worse cardiac and renal function in MET-free patients. Both cardiac as well as renal dysfunction are associated with higher GDF-15 levels^40^. MET-treated patients have increased ketone body beta-hydroxybutyrate, a metabolic substrate that is readily utilized by failing heart and that may have favorable effects on bioenergetics^41^. Infusion of ketone bodies in HFrEF patients was shown to improve cardiac output and LV-ejection fraction^42^. Interestingly, despite patients on MET had lower neurohumoral activation, we observed higher levels of stress hormone glucagon in MET-treated patients. Is was shown that MET administration to prediabetic subjects resulted in an increase of glucagon^43^. Higher glucagon in MET-treated patients may be protective against hypoglycemia that is a feared complication of DM treatment and was linked to arrhythmias and increased mortality^44^.

Although QoL was independent of DM status in our study, we have observed a better QoL in MET-treated DM patients and MET was significantly associated with QoL also in multivariable linear regression suggesting its independent effect on QoL. To our best knowledge, this is the first study that analyzed the QoL with respect to MET treatment using a validated tool^45^. It has been recently demonstrated that QoL in HF patients is driven by HF itself, not by associated comorbidities^46^. MET-induced improvement in myocardial efficiency^29^ suggests that the effect of MET on QoL in HF is rather due to an improvement in HF, not due to improvement in blood glucose control.

Beneficial effects of MET documented in high risk advanced HF population suggest that MET should be more widely used in management of HF. Even in studies with SGLT2 inhibitors in HF patients, a substantial proportion of DM+ patients were treated with biguanides (metformin). In DAPA-HF trial, 41.8% of patients had DM and 51% of DM+ patients were treated by biguanides (predominantly MET)^47^. EMPEROR-Reduced trial reports also a 49.8% prevalence of DM and 46.4% of DM+ patients were treated with biguanides^48,49^. Our data strongly suggest that MET should be a frontline drug for the treatment of diabetic patients with HFrEF. Combination therapy with MET and SGLT2i has been shown safe and efficacious in patients with DM^50^. Combined therapy of these patients with MET and SGLT2 inhibitors warrants further research.

### Limitations

Our study was performed in a heart center offering a complex cardiovascular program including MCS implantation and HTx. Since this could introduce bias related to the analysis of prognostic value, urgent HTx and MCS implantation were considered adverse outcomes, while the patients receiving non-urgent HTx were censored as having no adverse outcome on the day of transplantation^12^. In addition, it was a single-center study with a substantial predominance of males. Our study cohort included patients with advanced HFrEF, the results thus might not be fully applicable to patients with milder HF or to older patients. Data about HF re-hospitalizations were not available in all patients so this endpoint could have not been included in the analysis. The information about DM duration and MET exposure time before entering the study were not available in all subjects, therefore it was not possible to address these variables in the propensity-matching analysis. The cause of death was not available in all patients, so we were not able to distinguish between cardiovascular and non-cardiovascular mortality. QoL was analyzed only at baseline and is likely a result of a various time of preceding MET therapy; the time of MET treatment before baseline exam or during follow-up is unknown. Data about plasmatic MET concentration are not available; similarly, lactate was not measured. None of the patients was treated by sacubitril-valsartan or SGLT2 inhibitors. Therefore, it is impossible to analyze potentially additional effect of MET and these agents. Only a subset of patients had serial echocardiographic examinations, so it was not possible to analyze the impact of MET-treatment of cardiac reverse remodeling.

## Conclusion

Metformin treatment in advanced HFrEF patients with DM is associated with better outcome by mechanisms beyond the improvement of blood glucose control. Metformin should stay among frontline drugs for the management of HFrEF patients with DM.

## Supplementary Information

Supplementary Information.

Supplementary Figure 1.

Supplementary Figure 2.

Supplementary Figure 3.

Supplementary Figure 4.

Supplementary Table 1.

Supplementary Table 2.

Supplementary Table 3.

Supplementary Table 4.

## Data Availability

The datasets analyzed during the current study are available from the corresponding author on reasonable request (jan.benes@ikem.cz).
