# Supplementary material for: Metformin treatment is associated with improved outcome in patients with diabetes and advanced heart failure (HFrEF)
Source: Sci Rep. 2022 Jul 29;12:13038. doi: 10.1038/s41598-022-17327-4 (PMC9338272; doi:10.1038/s41598-022-17327-4)
Supplement: Supplementary file 1 — Supplementary Information. [file 41598_2022_17327_MOESM1_ESM.docx]

**Methods**
**Study subjects**

Patients with stable HFrEF (LVEF <40%) were enrolled in the study between 2008 and 2016 in a prospectively defined registry. Patients had to have at least 6-month history of HFrEF and had to receive stable medical therapy for at least three months. Subjects with potentially reversible LV dysfunction (planned valve surgery, revascularization, or tachycardia-induced cardiomyopathy) were excluded. Patients were followed until July 2019. DM was diagnosed according to current recommendation ^1^ either as a patient´s history of known DM, or Hb1Ac ≥48 mmol/mol (≥ 6.5% NGSP/DCCT) if DM was undetected before the study enrollment. The investigation conforms with the principles outlined in the Declaration of Helsinki, the study protocol was approved by the Institutional Ethics Committee and all subjects signed an informed consent.

At the study enrollment, patients completed a Minnesota Living with Heart Failure Questionnaire (MLHFQ) and had anthropometric tests and underwent an echocardiographic study (Vivid-7; General Electric, Milwaukee, Wisconsin). LV function and dimensions were measured according to recommendations.^2^ Mitral and tricuspid regurgitations were assessed semiquantitatively and expressed in 3 grades (mild, moderate, significant).
Right ventricular dysfunction was quantified (0 to 3) in an apical 4-chamber view by using tricuspid annular systolic excursion (M-mode TAPSE)^3^ and tissue systolic velocity (Sm)^4^ with the following cutoffs: RVD0, normal: TAPSE >20 mm, Sm >12 cm/s; RVD1, mild impairment: TAPSE 16 to 20 mm, Sm 9 to 12 cm/s; RVD2, moderate: TAPSE 10 to 15 mm, Sm 6 to 9 cm/s; and RVD3, severe: TAPSE severe: TAPSE <10 mm, Sm <6 cm/s. In case of disagreement of criteria, qualitative visual estimation of RV motion in apical 4-chamber was also taken into account.

**Follow-up**
The study was conducted in a tertiary cardiac center offering heart transplantation program and implantation of mechanical circulatory the support. Therefore, an adverse outcome was defined as the combined endpoint of death, urgent heart transplantation (HTx) or mechanical circulatory support (MCS) implantation. ^5^ As the time to non-urgent HTx reflects primarily donor availability rather than recipient’s condition, patients who received a non-urgent HTx were censored as having no adverse event at the day of HTx.
 **Laboratory assessment**Blood was collected into serum separator tubes and EDTA-containing tubes upon patient enrollment. Basic biochemical parameters were assessed at the Institute for Clinical and Experimental Medicine. BNP was measured on the ARCHITECT analyzer (Abbott Diagnostics, Abbott Park, Illinois) using a chemiluminescent immunoassay. HbA1c was measured on Tosoh analyser G8 (Tosoh Corporation, Tokyo, Japan) by automated high performance liquid chromatography. High-sensitivity troponin T was measured on the Cobas analyser e601 (Roche Diagnostics, Mannheim, Germany). Estimated glomerular filtration rate (eGFR) was calculated using CKD-EPI 2009 equation.
Insulin and C-peptide were measured using IRMA kits (Beckman Coulter, Prague, Czech Republic) and glucagon using RIA kit (EMD Millipore Corporation, St. Louis, Missouri, USA).
GDF-15 was measured using the Quantikine Human GDF-15 Immunoassay (R&D Systems Inc, Minneapolis, MN). Insulin resistance was estimated by HOMA-IR (homeostatic model assessment of insulin resistance) calculated as fasting glucose (mmol/l) x fasting immunoreactive insulin (microIU/ml) /22.5.^6^

**Statistical analysis**
Data are presented as mean ± standard deviation, median with interquartile ranges (IQRs), or frequency (percent). Unpaired t-test or Mann-Whitney test were used to compare continuous variables between groups as appropriate. Kolmogorov–Smirnov test was used to evaluate Gaussian distribution. Chi-square test was employed to compare categorical variables. The effect of biomarker concentration on prognosis was tested using univariate and multivariable Cox model. Event-free survival of patients was analyzed by Kaplan-Meier analysis with log-rank test comparison between groups.
Propensity score matching was used to account for differences in characteristics of patients with and without MET. The propensity score for each patient was calculated using a multivariable logistic regression model in which the MET use was regressed on 17 characteristics (see Results) that might influence the selection of MET therapy or that have been shown to influence prognosis of patients with advanced HF. Subjects were matched on the logit of the propensity score using 1:1 greedy nearest-neighbor matching with a caliper distance of 0.2 times the SD of the logit of the propensity score. Success of matching was assessed by computing the standardized mean difference of each covariate. To compare mortality between the 2 matched groups we have used the McNemar test and matched pairs stratified Cox proportional hazards model with a robust variance estimator. The proportional hazard assumption was tested and fulfilled. All tests were 2-sided, and p values <0.05 were considered significant. Calculations were performed using JMP 11 (SAS Institute Inc., Cary, NC) and R (Vienna, Austria).

**References**

1 Cosentino, F. *et al.* 2019 ESC Guidelines on diabetes, pre-diabetes, and cardiovascular diseases developed in collaboration with the EASD. *European heart journal* **41**, 255-323, doi:10.1093/eurheartj/ehz486 (2020).

2 Lang, R. M. *et al.* Recommendations for chamber quantification. *European journal of echocardiography : the journal of the Working Group on Echocardiography of the European Society of Cardiology* **7**, 79-108, doi:10.1016/j.euje.2005.12.014 (2006).

3 Kaul, S., Tei, C., Hopkins, J. M. & Shah, P. M. Assessment of right ventricular function using two-dimensional echocardiography. *American heart journal* **107**, 526-531, doi:10.1016/0002-8703(84)90095-4 (1984).

4 Meluzín, J. *et al.* Pulsed Doppler tissue imaging of the velocity of tricuspid annular systolic motion; a new, rapid, and non-invasive method of evaluating right ventricular systolic function. *European heart journal* **22**, 340-348, doi:10.1053/euhj.2000.2296 (2001).

5 Aaronson, K. D. *et al.* Development and prospective validation of a clinical index to predict survival in ambulatory patients referred for cardiac transplant evaluation. *Circulation* **95**, 2660-2667, doi:10.1161/01.cir.95.12.2660 (1997).

6 Matthews, D. R. *et al.* Homeostasis model assessment: insulin resistance and beta-cell function from fasting plasma glucose and insulin concentrations in man. *Diabetologia* **28**, 412-419, doi:10.1007/bf00280883 (1985).

**Online supplement figure legends

Figure S1.** Consolidated Standards of Reporting Trials (CONSORT) diagram. **Figure S2**. Event-free survival of DM-free and DM+ patients **Figure S3.** DM treatment. The information about DM treatment was missing in 3 patients. **Figure S4.** Distribution of MET daily dose.
 87 patients were treated with MET; MET daily dose was unknown in one patient.
