# Supplementary figures and images for: Metformin treatment is associated with improved outcome in patients with diabetes and advanced heart failure (HFrEF)
Source: Sci Rep. 2022 Jul 29;12:13038. doi: 10.1038/s41598-022-17327-4 (PMC9338272; doi:10.1038/s41598-022-17327-4)

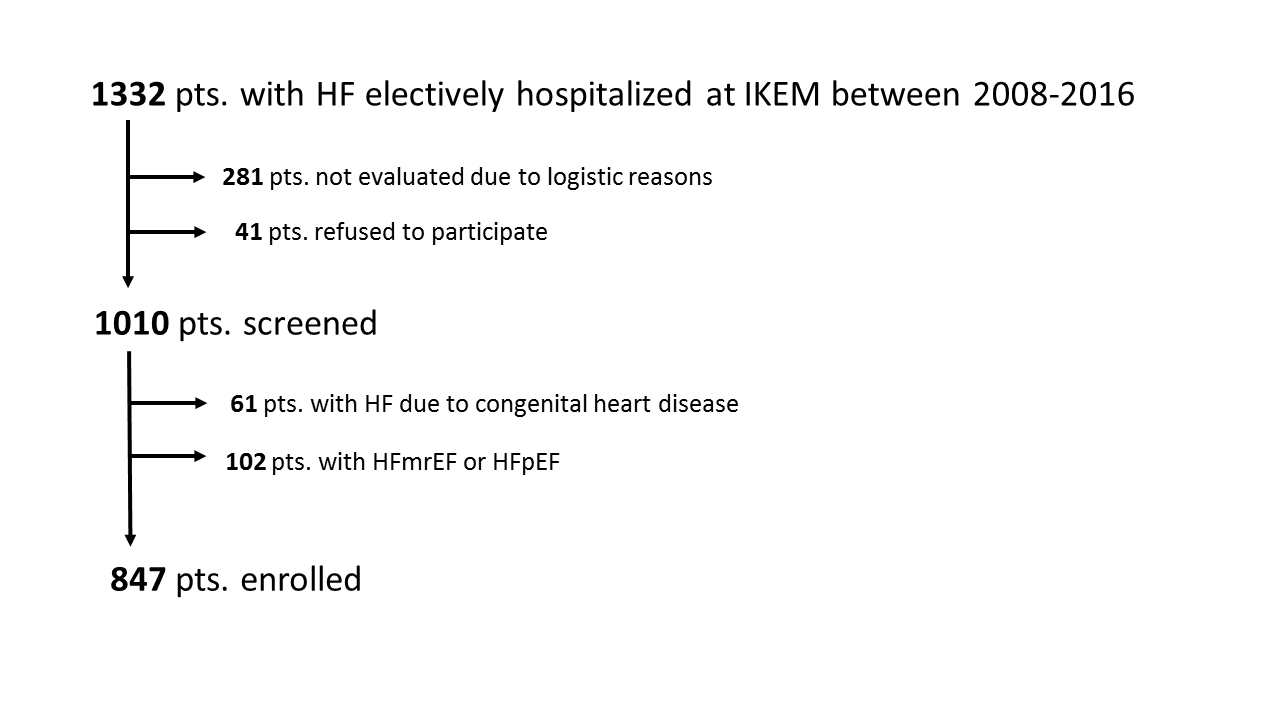

Supplement: Supplementary file 2 — Supplementary Figure 1. [file 41598_2022_17327_MOESM2_ESM.tif]

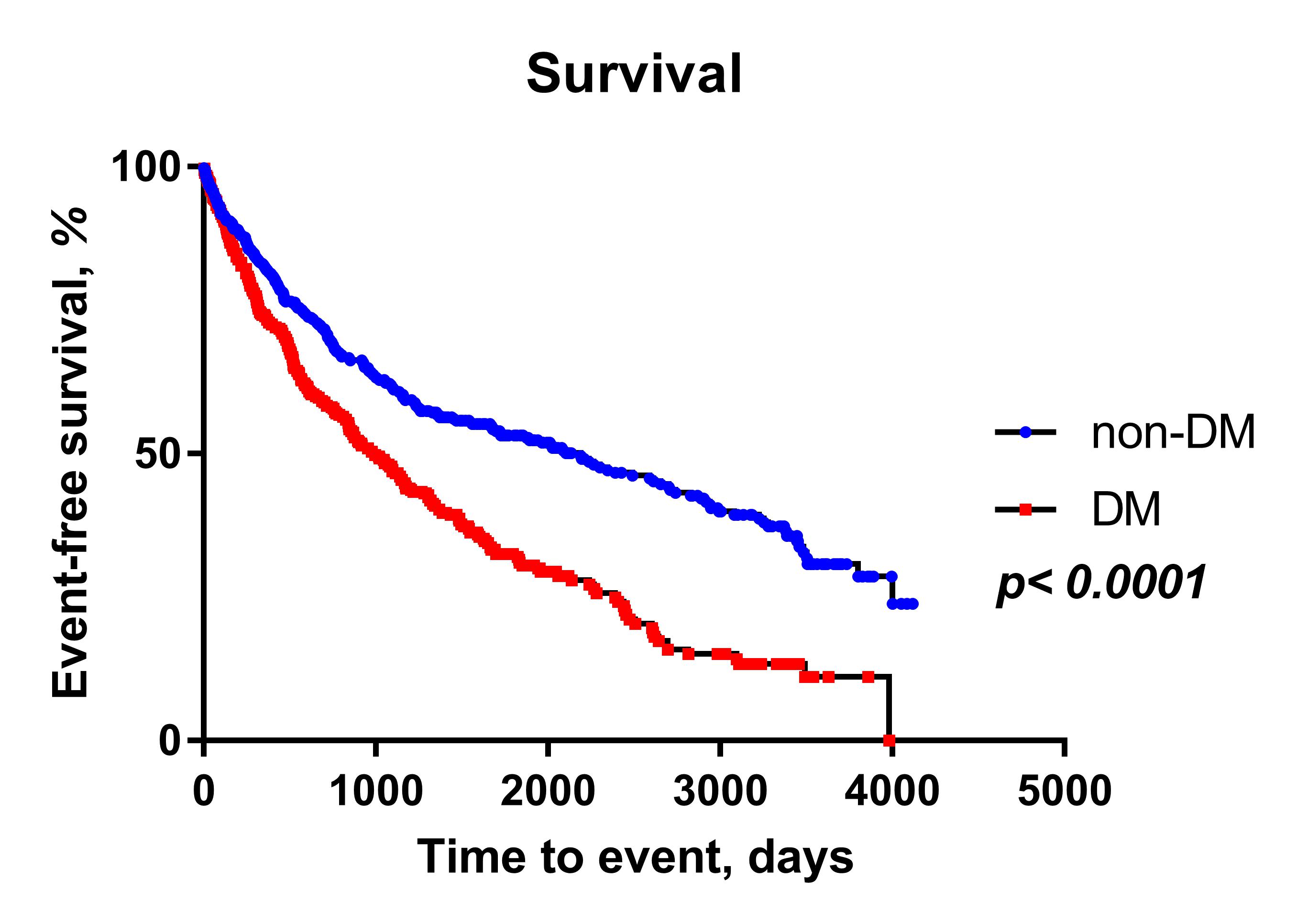

Supplement: Supplementary file 3 — Supplementary Figure 2. [file 41598_2022_17327_MOESM3_ESM.jpg]

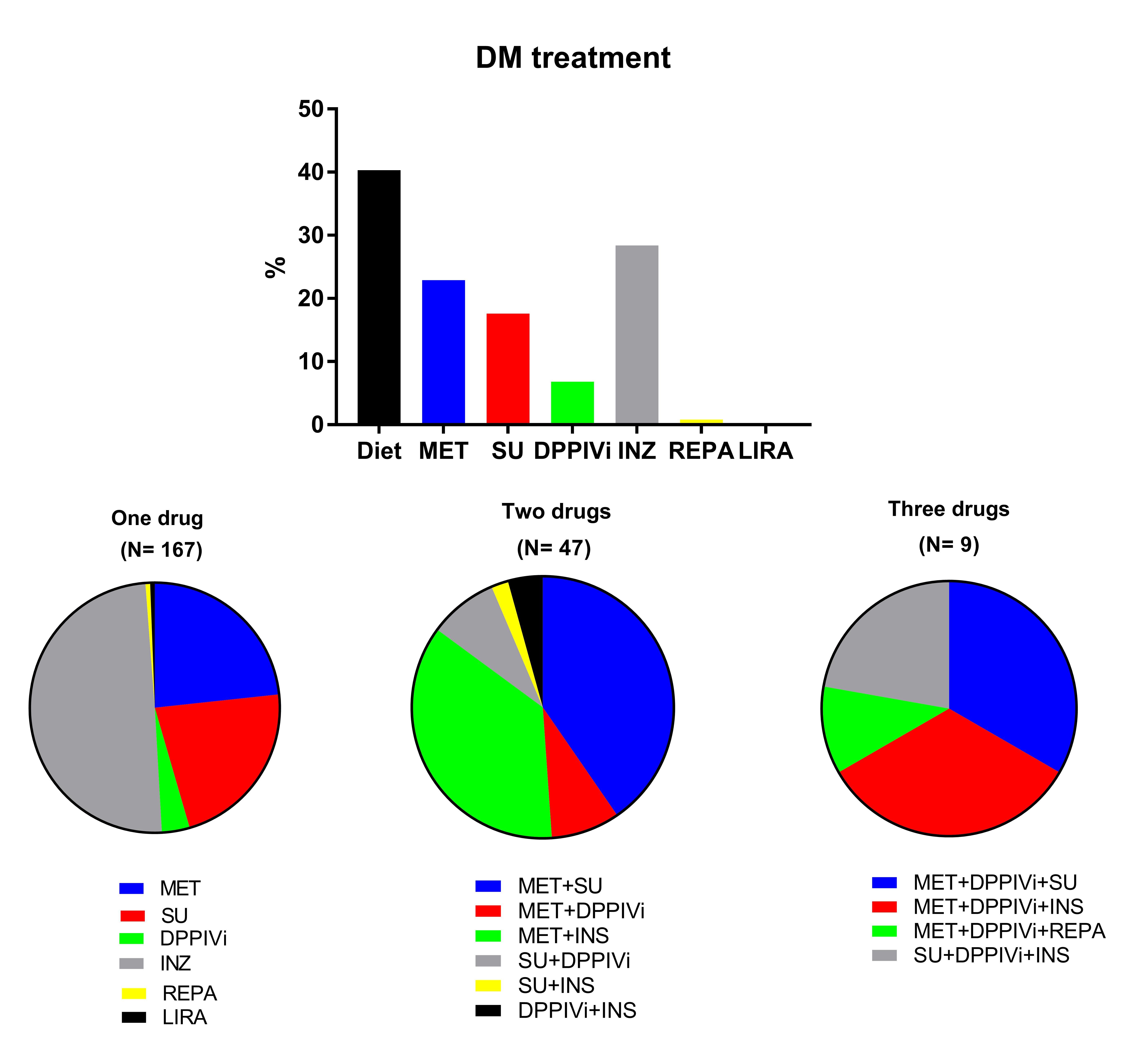

Supplement: Supplementary file 4 — Supplementary Figure 3. [file 41598_2022_17327_MOESM4_ESM.jpg]

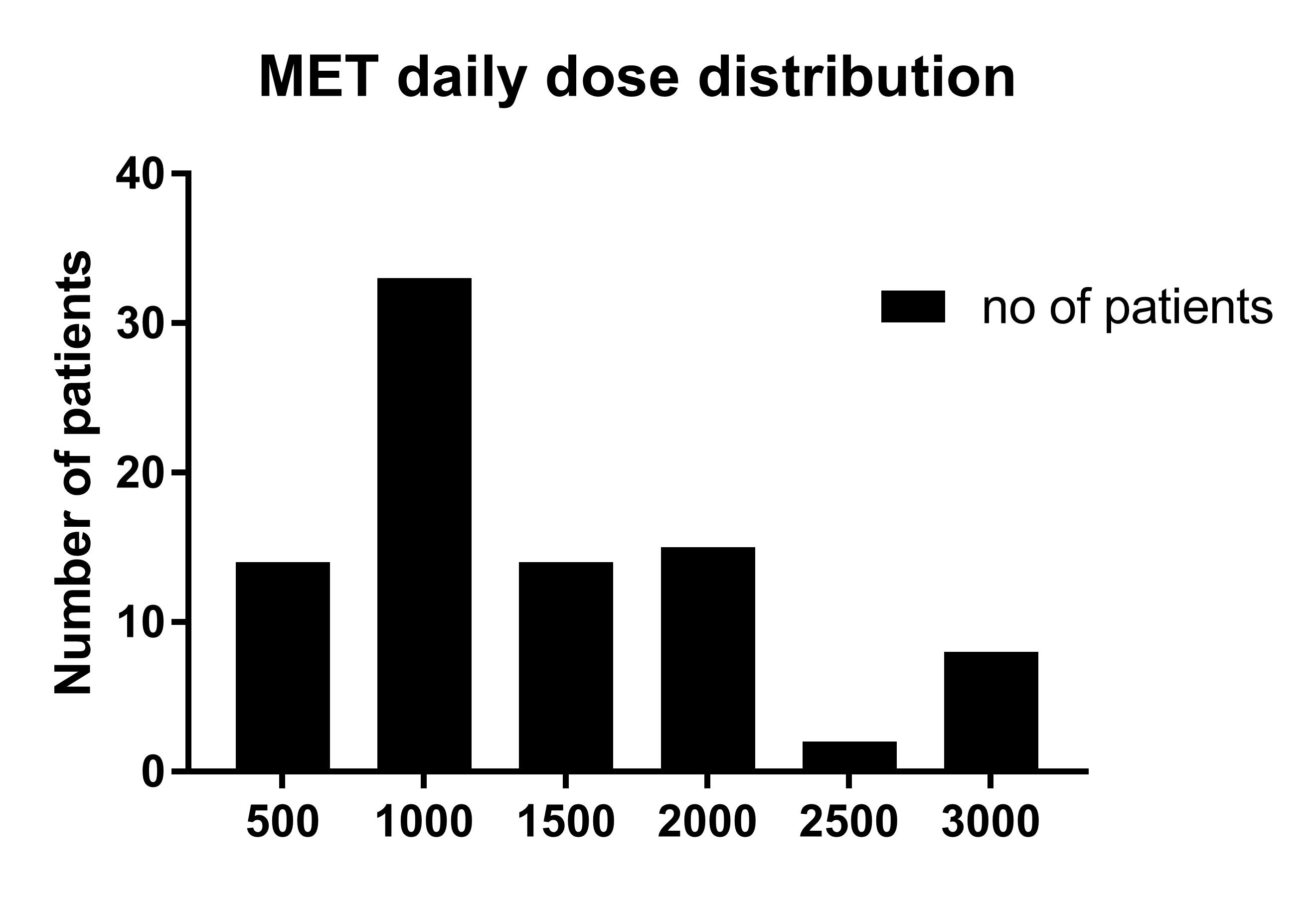

Supplement: Supplementary file 5 — Supplementary Figure 4. [file 41598_2022_17327_MOESM5_ESM.jpg]
