# Supplementary material for: Metformin treatment is associated with improved outcome in patients with diabetes and advanced heart failure (HFrEF)
Source: Sci Rep. 2022 Jul 29;12:13038. doi: 10.1038/s41598-022-17327-4 (PMC9338272; doi:10.1038/s41598-022-17327-4)
Supplement: Supplementary file 6 — Supplementary Table 1. [file 41598_2022_17327_MOESM6_ESM.docx]

**Supplementary table 1: DM treatment and quality of life**

MLHFQ - Minnesota living with heart failure questionnaire; SU – sulfonylurea.

|  | **No insulin** | **Insulin** |  |
| --- | --- | --- | --- |
| MLHFQ sum | 44 (28; 60) | 44 (29; 61) | 0.92 |
| MLHFQ somatic | 22 (12; 27) | 21 (12; 29) | 0.70 |
| MLHFQ emotional | 6 (2; 12) | 6 (3; 10) | 0.70 |
|  |  |  |  |
|  | **No SU derivatives** | **SU derivatives** |  |
| MLHFQ sum | 45 (29; 61) | 39 (22.5; 55.5) | 0.25 |
| MLHFQ somatic | 22 (13; 28) | 20 (10; 26) | 0.20 |
| MLHFQ emotional | 6 (2; 11) | 5 (1; 12) | 0.55 |
|  |  |  |  |
|  | **No DPPIV-inhibitors** | **DPPIV-inhibitors** |  |
| MLHFQ sum | 44 (29; 61) | 38 (14; 51) | 0.07 |
| MLHFQ somatic | 22 (13; 28) | 18 (6; 24) | 0.08 |
| MLHFQ emotional | 6 (2; 12) | 5 (0; 8) | 0.10 |

Treatment with insulin, SU derivatives or DPPIV-inhibitors was not associated with better QoL.
