# Supplementary material for: Metformin treatment is associated with improved outcome in patients with diabetes and advanced heart failure (HFrEF)
Source: Sci Rep. 2022 Jul 29;12:13038. doi: 10.1038/s41598-022-17327-4 (PMC9338272; doi:10.1038/s41598-022-17327-4)
Supplement: Supplementary file 7 — Supplementary Table 2. [file 41598_2022_17327_MOESM7_ESM.docx]

|  | r | P |
| --- | --- | --- |
| BNP *(ng.L^-1^)* | 0.006 | **<0.0001** |
| BMI *(kg.m^-2^)* | 0.82 | **0.0007** |
| eGFR *(ml.min^-1^.1.73m^-2^)* | 0.01 | 0.85 |
| MET *(present vs. absent)* | - 9.2 | **0.003** |
| SU derivatives *(present vs. absent)* | 1.2 | 0.73 |
| DPPIVi *(present vs. absent)* | -7.9 | 0.12 |
| Insulin *(present vs. absent)* | -1.4 | 0.62 |

**Supplementary table 2: Variables associated with MLHFQ score
(DM patients only, n= 380)**


MLHFQ - Minnesota living with heart failure questionnaire
BMI and BNP and MET, but not other glucose-lowering agents, were associated with QoL (MLHFQ score) in multivariable linear regression model (r^2^ for all variables = 0.11).
