# Supplementary material for: Metformin treatment is associated with improved outcome in patients with diabetes and advanced heart failure (HFrEF)
Source: Sci Rep. 2022 Jul 29;12:13038. doi: 10.1038/s41598-022-17327-4 (PMC9338272; doi:10.1038/s41598-022-17327-4)
Supplement: Supplementary file 8 — Supplementary Table 3. [file 41598_2022_17327_MOESM8_ESM.docx]

|  | Univariable analysis | | |
| --- | --- | --- | --- |
|  | HR | 95% CI | p |
| MET treatment *(present vs. absent)* | 0.57 | 0.41- 0.78 | **0.0003** |
| Insulin treatment *(present vs. absent)* | 0.97 | 0.74- 1.26 | 0.82 |
| SU derivatives treatment *(present vs. absent)* | 0.95 | 0.68- 1.28 | 0.61 |
| DPPIV-inhibitors treatment *(present vs. absent)* | 0.73 | 0.41- 1.21 | 0.24 |

**Supplementary table 3: DM treatment and outcome**Cox proportional hazard model. Treatment with MET but not with other PAD or insulin was associated with lower risk of adverse event. HR, hazard ratio. CI, confidence interval.
