# Supplementary material for: Metformin treatment is associated with improved outcome in patients with diabetes and advanced heart failure (HFrEF)
Source: Sci Rep. 2022 Jul 29;12:13038. doi: 10.1038/s41598-022-17327-4 (PMC9338272; doi:10.1038/s41598-022-17327-4)
Supplement: Supplementary file 9 — Supplementary Table 4. [file 41598_2022_17327_MOESM9_ESM.docx]

| **variable** | **P for interaction** |
| --- | --- |
| NYHA functional class | 0.65 |
| LVEF | 0.42 |
| RV dysfunction grade | 0.43 |
| BNP | 0.35 |
| eGFR | 0.20 |
| ACEi/ARB treatment | 0.31 |
| beta-blocker treatment | 0.93 |
| ICD | 0.50 |
| CRT | 0.28 |

**Supplementary table 4: Interaction between MET treatment and HF-related and renal parameters**
